# Implementation and evaluation of a paediatric nurse-driven sedation protocol in a paediatric intensive care unit

**DOI:** 10.1186/s13613-017-0256-7

**Published:** 2017-03-24

**Authors:** Lélia Dreyfus, Etienne Javouhey, Angélique Denis, Sandrine Touzet, Fabienne Bordet

**Affiliations:** 1 Service de réanimation pédiatrique, Hospices Civils de Lyon - Hôpital Femme Mère Enfant, 59, Boulevard Pinel, 69500 Bron, France; 2 Université Claude-Bernard Lyon 1, 69008 Lyon, France; 3 Pôle information médicale évaluation recherche, Hospices Civils de Lyon, 162 avenue Lacassagne Bâtiment A, 69003 Lyon, France; 4 HESPER EA 7425, Université Claude Bernard Lyon 1, 69008 Lyon, France

**Keywords:** PICU, Nurse-driven sedation protocol, Mechanical ventilation, Withdrawal symptoms, COMFORT-B score, Opioid, Benzodiazepine, Evaluation, Feasibility

## Abstract

**Background:**

Optimal sedation and analgesia is a challenge in paediatric intensive care units (PICU) because of difficulties in scoring systems and specific metabolism inducing tolerance and withdrawal. Excessive sedation is associated with prolonged mechanical ventilation and hospitalisation. Adult and paediatric data suggest that goal-directed sedation algorithms reduce the duration of mechanical ventilation. We implemented a nurse-driven sedation protocol in a PICU and evaluated its impact.

**Methods:**

We conducted a before and after protocol implementation study in a population of children aged 0–18 years who required mechanical ventilation for at least 24 h between January 2013 and March 2015. After the protocol implementation in January 2014, nurses managed analgesia and sedation following an algorithm that included the COMFORT behaviour scale (COMFORT-B). Duration of mechanical ventilation was the primary outcome; secondary outcomes were total doses and duration of medications, PICU length of stay, incidence of ventilator-associated pneumonia, and occurrence of withdrawal symptoms. Pre–post analysis followed with segmented regression analysis of interrupted time series was used to assess the effect of protocol.

**Results:**

A total of 200 children were analysed, including 107 before implementation and 93 children after implementation of the protocol. After implementation of the protocol, the total number of COMFORT-B scores per day of mechanical ventilation significantly increased from 3.9 ± 2.5 times during the pre-implementation period to 6.6 ± 3.5 times during the post-implementation period (*p* < 10^−3^). Mean duration of mechanical ventilation tended to be lower in the post-implementation period (8.3 ± 7.3 vs 6.6 ± 5.6 days, *p* = 0.094), but changes in either the trend per trimester from pre-implementation to post-implementation (*p* = 0.933) or the immediate change after implementation (*p* = 0.923) were not significant with segmented regression analysis. No significant change between pre- and post-implementation was shown for total dose of sedatives, withdrawal symptoms, agitation episodes, or unplanned endotracheal extubations.

**Conclusions:**

These results were promising and suggested that implementation of a nurse-driven sedation protocol in a PICU was feasible. Evaluation of sedation and analgesia was better after the protocol implementation; duration of mechanical ventilation and occurrence of withdrawal symptoms tended to be reduced.

**Electronic supplementary material:**

The online version of this article (doi:10.1186/s13613-017-0256-7) contains supplementary material, which is available to authorized users.

## Background

Management of analgesia and sedation is an integral component of medical care for critically ill children. Its role is to assure the comfort and safety of a patient undergoing painful treatments and technical procedures. It can also be, particularly in situations like acute respiratory distress syndrome (ARDS) or acute brain injury, a full processing treatment [1].

Optimal sedation is described as a patient under sedation who can easily be woken up and who can undergo medical care and procedures. On both sides of this optimal state are the states of “oversedation” and “undersedation”, both with major drawbacks. Excessive sedation is associated with poor outcomes like prolonged mechanical ventilation, longer hospitalisation, more nosocomial infections, and more frequent withdrawal symptoms [2–4]. Insufficient sedation involves risks of agitation and complications like unplanned extubation or catheter removal.

In a literature review in the paediatric population, Vet et al. [5] showed that optimal sedation is obtained in only 60% of children and that “oversedation” is more frequent than “undersedation” (30% versus 10%). Recommendations in paediatric populations are rare, but most of the time the combination of an opioid and a sedative is used. Playfor et al. [6] recommended using morphine or fentanyl with midazolam in a continuous intravenous perfusion. A survey published in Critical Care Medicine by Kudchadkar et al. [7] done in paediatric intensive care units (PICU) all over the world reported that 72% of the centres used opioids and benzodiazepines together and only 2% use ketamine or propofol.

Good management of sedation and analgesia is partly based on good evaluation and requires validated, simple, and reproducible scores. Many different scores exist to evaluate pain in children, but only the COMFORT scale is validated in children under sedation. Derived from the COMFORT scale, the COMFORT behaviour scale (COMFORT-B) was published by Ista et al. [8]. This scale excludes the physiologic items from the COMFORT scale and retains only the behavioural items. It showed very good correlation with the Nurse Interpretation Score of Sedation. In Kudchadkar’s survey [7], even so only 42% of PICUs routinely used an objective sedation score.

Sedation and analgesia is particularly challenging in children with regards to a specific metabolism because tolerance and withdrawal phenomena are frequently observed in cases of prolonged administration [9]. Many studies have proved that continuous intravenous perfusion of drugs is associated with longer mechanical ventilation [2, 3]. Recent years have seen emerging techniques of goal-directed sedation. These include daily interruption of sedatives and nurse-driven protocols. In adults, Kress et al. [10] evaluated daily interruption of sedatives and showed a significant reduction in the duration of mechanical ventilation, length of hospital stay, and cumulative doses of midazolam and opioids. Nurse-driven sedation and analgesia protocols were evaluated in adults and, despite interesting results [11–13], a Cochrane review in 2015 concluded that there was insufficient evidence to evaluate the effectiveness of protocol-directed sedation [14]. In paediatric units, goal-directed sedation protocols were also developed but few studies were found in the literature, with unclear results [15–17]. Recently, a randomised controlled trial among children admitted in PICU for acute lung injury showed no difference in duration of ventilation regardless of sedation protocol or usual care implementation [18]. Daily interruption of sedation was also studied in PICU: Gupta et al. [19] and Verlaat et al. [20] showed a significant reduction in duration of mechanical ventilation, PICU length of stay, and duration of benzodiazepine perfusion with a daily sedation interruption protocol. However, more recently a RCT including 129 patients [21] under sedation protocol assessed the impact of daily sedation interruption in critically ill children and no improvement of clinical outcomes was found; an increased mortality was even found in the group with daily sedation interruption without any evidence of a relationship.

The aim of this study was to evaluate the impact of a nurse-driven sedation protocol implemented at a PICU on the duration of mechanical ventilation, total dose of sedatives, length of stay in the PICU, incidence of ventilator-associated pneumonia, and occurrence of withdrawal symptoms.

## Patients and methods

### Study location and population

The study was set up between January 2013 and March 2015 at a 23-bed medical–surgical PICU of a university-affiliated teaching hospital, counting 10 intensivists and 65 nurses. Newborns and post-cardiac surgery patients were not admitted in our PICU. The nurse/patient ratio was 1:2 in the two periods of the study. Mechanically ventilated patients aged from 0 to 18 years receiving sedation for more than 24 h were included in the study. Patients who were transferred from another PICU where sedative drugs had already been initiated and patients with tracheostomy were excluded from the study. No written ventilation weaning protocol existed in the PICU. Spontaneous breathing tests with T-piece, pressure support or pressure support and CPAP before mechanical ventilation weaning were performed as stated by the French recommendations [22]. Weaning criteria are checked at least twice a day. Neither nurses nor respiratory therapists or physiotherapists are usually involved in ventilation weaning in France.

### Study design

We conducted a before and after protocol implementation study with series of measurement over time interrupted by an intervention. Such design is an alternative approach used to evaluate the effects of any intervention, when randomised controlled trials are infeasible or identification of a control group impractical [23, 24]. Data collection took place during a 12-month pre-implementation period between January 2013 and December 2013 and during an 11-month post-implementation period between May 2014 and March 2015. The post-implementation data collection period started 4 months after the implementation of the protocol in January 2014. During this 4-month lag, the protocol was introduced and explained to all the nurses and medical staff and no data was collected.

### Drugs

During all the study period, the standard therapy was the association of an opioid (sufentanil initiated at 0.2–0.3 µg/kg/h) and a hypnotic (midazolam initiated at 0.05–0.1 mg/kg/h and/or ketamine initiated at 1 mg/kg/h), both in continuous intravenous perfusions, with allowed boluses. During continuous sedation weaning, other treatments could be administered such as methadone, hydroxyzine, clonidine, and long half-life benzodiazepines or neuroleptics according to the PICU guidelines.

### Instrument

COMFORT scale and COMFORT behaviour scale are the only validated sedation scales for mechanically ventilated children. COMFORT-B scale includes 6 behavioural items which are state of awakening, levels of agitation, spontaneous ventilation, characteristics of movements, muscular tone, and faces. Contrary to original COMFORT scale, it does not contain physiological items.

Target ranges are inspired from the study of Ista et al. [7] that suggests that a COMFORT-B score between 6 and 10 is associated with excessive sedation, a score between 18 and 30 with insufficient sedation, and a score between 11 and 17 with adequate sedation.

To evaluate withdrawal symptoms, Franck et al. [25] described the Withdrawal Assessment Tool-1 (WAT-1) and proved that it had a good specificity and sensibility to predict withdrawal symptoms. It was later widely validated by the RESTORE Investigative Team [26]. The WAT-1 score contains clinical observations before and after stimulation, the score ranges from 0 to 12, and a score ≥3 indicates withdrawal symptoms.

### Intervention plan

**Fig. 1:**
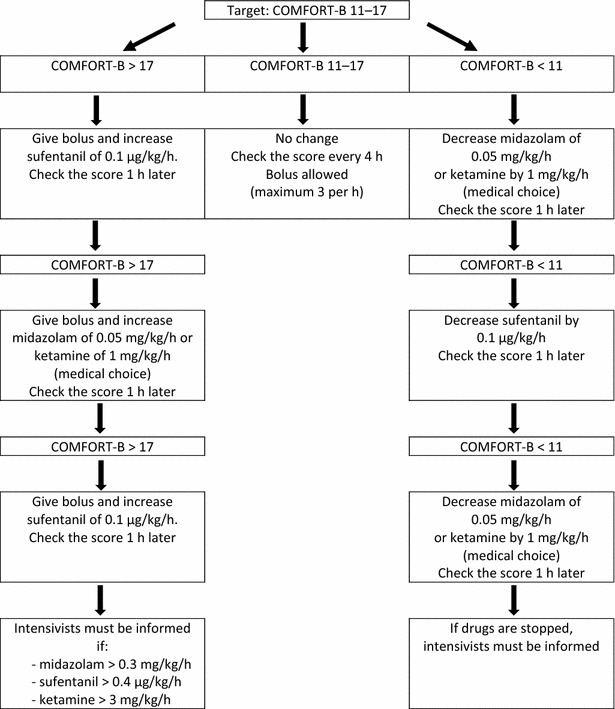
Nurse-driven sedation protocol

The implementation of the nurse-driven sedation protocol in the PICU started in January 2014 and was developed by two paediatric intensivists inspired by the experience of other PICU and local practices. During 4 months, training courses were organised by the two paediatric intensivists to teach and disseminate the protocol. Four sessions of 1 h were proposed, one for the 10 physicians and three for the 65 nurses divided into groups of about 20 people. During each session, general recalls were made about sedation, analgesia, evaluation with COMFORT-B scale; the protocol algorithm was detailed and explained; some clinical scenarios were discussed to illustrate the protocol use, and then, a questionnaire was left at the end of the session. A nurse working-group interested in management of pain was particularly involved and contributed to diffuse the protocol to all intensivists and nurses by training courses. When questions came up in daily routine, trained nurses and physicians were able to answer them and explain the algorithm. The sedation scoring system was the COMFORT-B scale. The algorithm is shown in Fig. 1; it was available at each PICU bed. Initial doses were chosen by the physicians, and then, all changes were made by the nurses with the aim of attaining an optimal range of analgesia and sedation, which was defined as values from 11 to 17 on the COMFORT-B score. The first evaluation with COMFORT-B was made 1 h after the therapeutics were introduced. If the score was greater than 17, a sign of insufficient sedation, the sufentanil dose was first raised; after 1 h, if the score was still greater than 17, a hypnotic, midazolam, or ketamine dose, depending on the physician’s choice, had to be raised. This was done alternately every hour until the score reached the optimal range. Once the COMFORT-B score was optimal, nurses assessed the score every four hours. If maximum doses of drugs were reached (midazolam 0.3 mg/kg/h, ketamine 3 mg/kg/h, or sufentanil 0.4 µg/kg/h), the treating physician had to be informed. On the other hand, if the score indicated oversedation (COMFORT-B less than 11), hypnotics and opioids were decreased alternately every hour. In addition to the continuous perfusion, boluses were allowed every hour, particularly during the nursing care. Bolus dose corresponded to the drug’s dose administered per hour: for example, if sufentanil infusion rate is 0.1 µg/kg/h, a bolus of 0.1 µg/kg could be given every hour. Before implementing this new strategy, all changes in the sedation and analgesia were evaluated by the physicians, which lengthened the period between the recognition of inadequate sedation and the adaptation of the therapeutics.

Patients who needed deeper sedation for medical reasons (for example, those with acute lung or brain injury) had a lower target score on the COMFORT-B (<11). Sedation and analgesia were then modified by the physicians, and the protocol was not applied. Also these patients were still included in the study, as all patients receiving more than 24 h of sedation.

### Measures

The primary outcome measure was duration of mechanical ventilation (days), and secondary outcome parameters were cumulative doses of drugs including continuous perfusion and boluses (unit/kg), duration of drug perfusion (h), duration of PICU stay (days), occurrence of withdrawal symptoms (evaluated by WAT-1 score), and incidence of ventilator-associated pneumonia (defined by the CDC criteria [27] and expressed in patients by 1000 days of mechanical ventilation). We were also interested by the impact of the protocol on evaluation of sedation and analgesia by comparing number of assessments by COMFORT-B scores per day of ventilation and number of adequate (COMFORT-B scores in the optimal range) and inadequate scores.

Clinical, epidemiologic, and demographic data were also collected including age, weight, medical histories, reason for admission, unplanned endotracheal extubation, severity score (PELOD), and death.

### Data collection

All data were extracted from patients’ computerised medical charts (ICCA^®^, Philips IntelliSpace Critical Care and Anesthesia).

### Statistical analysis

With about 100 children per period, a pre- versus post-implementation test of means would have 80% power to detect a mean reduction in the mechanical ventilation of 2 days assuming a standard deviation of 5 and alpha = 5%.

Means and standard deviations were reported for continuous variables, and percentages and frequencies for categorical variables. First, outcomes were compared between pre- and post-implementation periods using Wilcoxon’s test for continuous variables and Chi-square test for categorical variables. Comparison in duration of mechanical ventilation was controlled for age and reason of admission.

In a second step, to control for secular trends, we used segmented regression analyses of interrupted time series to assess the effect of the implementation of the nurse-driven sedation protocol on outcomes [28, 29]. We estimated the time trend in outcomes before the implementation, the change in trend after the implementation, and the change in level at the initiation of the nurse-driven sedation protocol on outcomes. The pre- and post-implementation period was divided into 4 periods of 3 months. Outcomes were aggregated into mean in 3-month intervals, the trimester representing the unit of analysis. An indicator variable was used to define the implementation of the nurse-driven sedation protocol, with a value of 0 given to the trimesters before implementation and a value of 1 given to the trimesters after implementation (beginning in May 2014). Model parameters included intercept, time trend before implementation, level change immediately after implementation, and change in time trend after implementation. The model is presented in “Appendix”. A *p* value <0.05 was regarded as statistically significant. The segmented regression analysis was not performed for withdrawal symptoms and ventilator-associated pneumonia because of the few events occurred over the study period. Statistical analyses were performed with SAS software (SAS Institute, Cary, NC, version 9.3).

## Results

### Patients’ characteristics

A total of 104 patients were enrolled during the 12-month pre-implementation period and 93 patients during the 11-month post-implementation period. Patients’ characteristics are presented in Table 1. There were no significant differences between study periods except for the type of admission (*p* = 0.039). In the pre-implementation period, 24% of patients were admitted for surgical cause and 38% during the post-implementation period.

**Table 1:** Patient characteristics over the study period

	Pre-implementation *n* = 104	Post-implementation *n* = 93	*p* value
Male, *N* (%)	61 (59)	57 (61)	0.706
Age at admission (years), mean (SD)	4.9 (5.4)	5.2 (5.3)	0.607
Median [Q1–Q3]	2.2 [0.4–9.6]	3.2 [0.4–9.7]	
Weight at admission (kg), mean (SD)	20.3 (18.9)	20.7 (17.8)	0.660
Reason for admission, *N* (%)*			
Surgical cause	25 (24)	35 (38)	0.039
Non-surgical cause	79 (76)	58 (62)	0.131
Neurology	41 (39)	28 (27)	
Respiratory	29 (29)	22 (21)	
Sepsis	9 (9)	8 (8)	
Medical history, *N* (%)	18 (17)	15 (16)	0.825
Prematurity <34 weeks	8 (8)	4 (4)	0.267
Chronic respiratory failure	1 (1)	3 (3)	0.345
Congenital cardiopathy	5 (5)	6 (6)	0.616
Chronic cardiac failure	0 (0)	2 (2)	0.212
Encephalopathy	8 (8)	5 (5)	0.513
PELOD score, mean (SD)	18.8 (11.6)	17.5 (11.6)	0.746
Death, *N* (%)	18 (17)	13 (14)	0.522
Inotropic drugs, *N* (%)	75 (72)	67 (72)	0.991
Neuromuscular blockade, *N* (%)	38 (37)	33 (35)	0.878
Dialysis, *N* (%)	2 (2)	4 (4)	0.424
Unplanned extubation, *N* (%)	4 (4)	3 (3)	1.000

* Reason for admission: *p* = 0.039 is the *p* value of the difference between surgical and non-surgical admission diagnosis; *p* = 0.131 is the *p* value of the difference within the non-surgical admission diagnosis (neurology, respiratory, and sepsis)

### Primary outcome

As there were significantly more surgical patients in the post-implementation period (*p* = 0.039; Table 1), we compared the duration of mechanical ventilation and PICU length of stay according to the admission diagnosis. We found a significant shorter duration of mechanical ventilation in patient admitted for surgical reasons: 5.8 ± 5.3 vs 8.2 ± 6.9 days for non-surgical pathologies (*p* = 0.016). In the year prior to the implementation of the nurse-driven sedation protocol, the mean duration of mechanical ventilation was 8.3 ± 7.3 days and decreased to 6.6 ± 5.6 days after the implementation. The difference between the two periods was not significant (*p* = 0.094) (Table 2), even after controlling for age at admission and reason for admission (*p* = 0.129, Additional file 1).

**Table 2:** Duration of mechanical ventilation, PICU length of stay, and medication uses over the study period

	Pre-implementation *n* = 104	Post-implementation *n* = 93	*p* value*
Duration of mechanical ventilation (days)	5.6 (3.2–10.5)	4.8 (2.8–8.3)	0.094
PICU length of stay (days)	9.0 (5.0–15.2)	9.8 (4.9–14.5)	0.767
Sufentanil			
Daily dose (μg/kg/day)	5.6 (3.8–8.7)	6.3 (4.7–8.7)	0.185
Duration of administration (h)	99 (54–170)	78 (47–133)	0.097
Midazolam			
Daily dose (mg/kg/day)	3.2 (2.1–4.9)	2.9 (2.2–4.4)	0.223
Duration of administration (h)	112 (60–171)	89 (44–155)	0.149
Ketamine			
Daily dose (mg/kg/day)	46.1 (29.6–57.1)	41.9 (24.3–51.3)	0.067
Duration of administration (h)	110 (54–167)	94 (46–155)	0.574

Quantitative variables are expressed as median (quartiles Q1–Q3). *P* values were calculated using Wilcoxon’s test

### Secondary outcomes

No significant change was shown in the PICU length of stay or in the medication uses (Table 2).

The rate of withdrawal symptoms was 23% (24/104) in pre-implementation and decreased non-significantly to 14% (13/104) in post-implementation period (*p* = 0.103) without increasing additional drugs (Additional file 2). The intervention had no significant effect on the rate of ventilator-associated pneumonia (reduction from 14 to 10%, *p* = 0.309) and on the incidence of ventilator-associated pneumonia (reduction from 17.4 to 14.7 per 1000 ventilator days, *p* = 0.690).

### Evaluation of sedation and analgesia: scoring with the COMFORT-B scale (secondary outcomes)

**Fig. 2:**
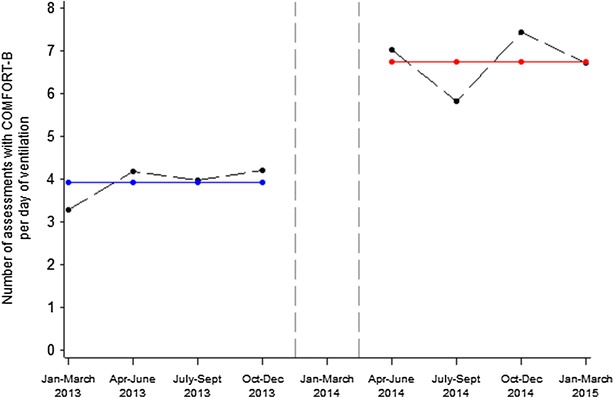
Mean number of COMFORT-B assessment per day on mechanical ventilation over time. Observed data are presented with *black dots,* and predicted data with segmented regression analysis are presented in *blue* for pre-implementation and *red* for post-implementation. *Vertical dashed lines* represent the 4-month implementation period

The mean number of time COMFORT-B was performed in the pre- and post-implementation groups was 3.9 ± 2.5 and 6.6 ± 3.5 (times per day of mechanical ventilation), respectively. The difference between the two periods was significant (*p* < 10^−3^). As shown in Fig. 2, segmented regression analysis confirmed the significant immediate increase of +2.8 times (SD = 0.4) in the number of assessment with the COMFORT-B scale per day on mechanical ventilation after protocol implementation (*p* = 0.0004).

**Fig. 3:**
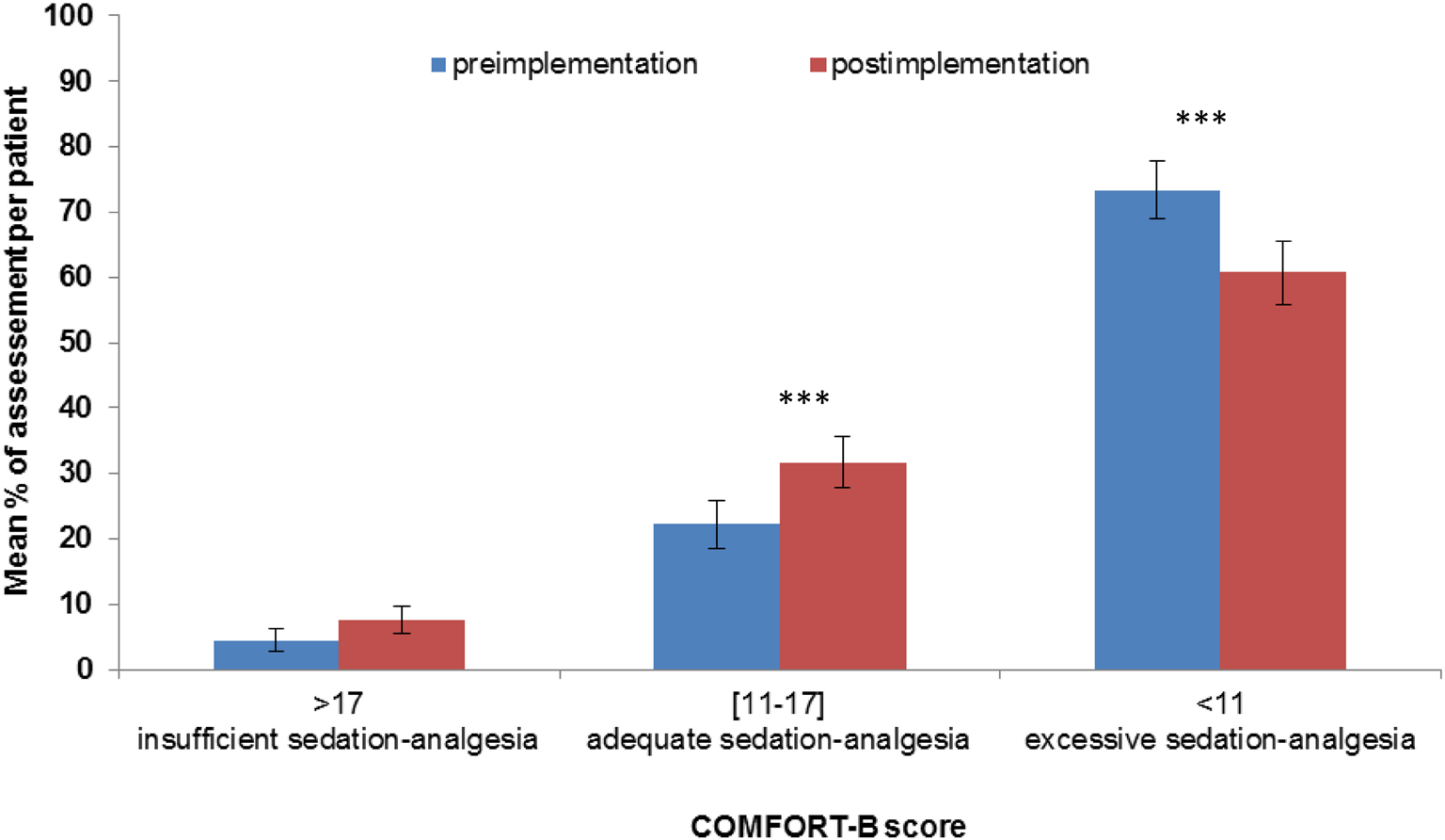
Distribution of COMFORT-B scores per level of sedation and analgesia before and after the protocol implementation. *Bars* represent 95% confidence interval, ***p < 0.001

The median COMFORT-B score per patient was 8 (range 6–19) during the pre-implementation period and 9.5 (range 6–15.5) during the post-implementation period. The difference between the two periods was significant (*p* = 0.002). Figure 3 shows the distribution of COMFORT-B scoring per patient for each period. The mean number of adequate (scores between 11 and 17) levels of sedation and analgesia was significantly greater after protocol implementation (22.2 vs 31.7%, *p* < 10^−3^), whereas the mean number of excessive (scores < 11) levels of sedation and analgesia decreased significantly from 73.3% in the pre-implementation to 60.7% in the post-implementation period (*p* < 10^−3^).

### Segmented regression analysis (additional analyses for primary and secondary outcomes)

**Fig. 4:**
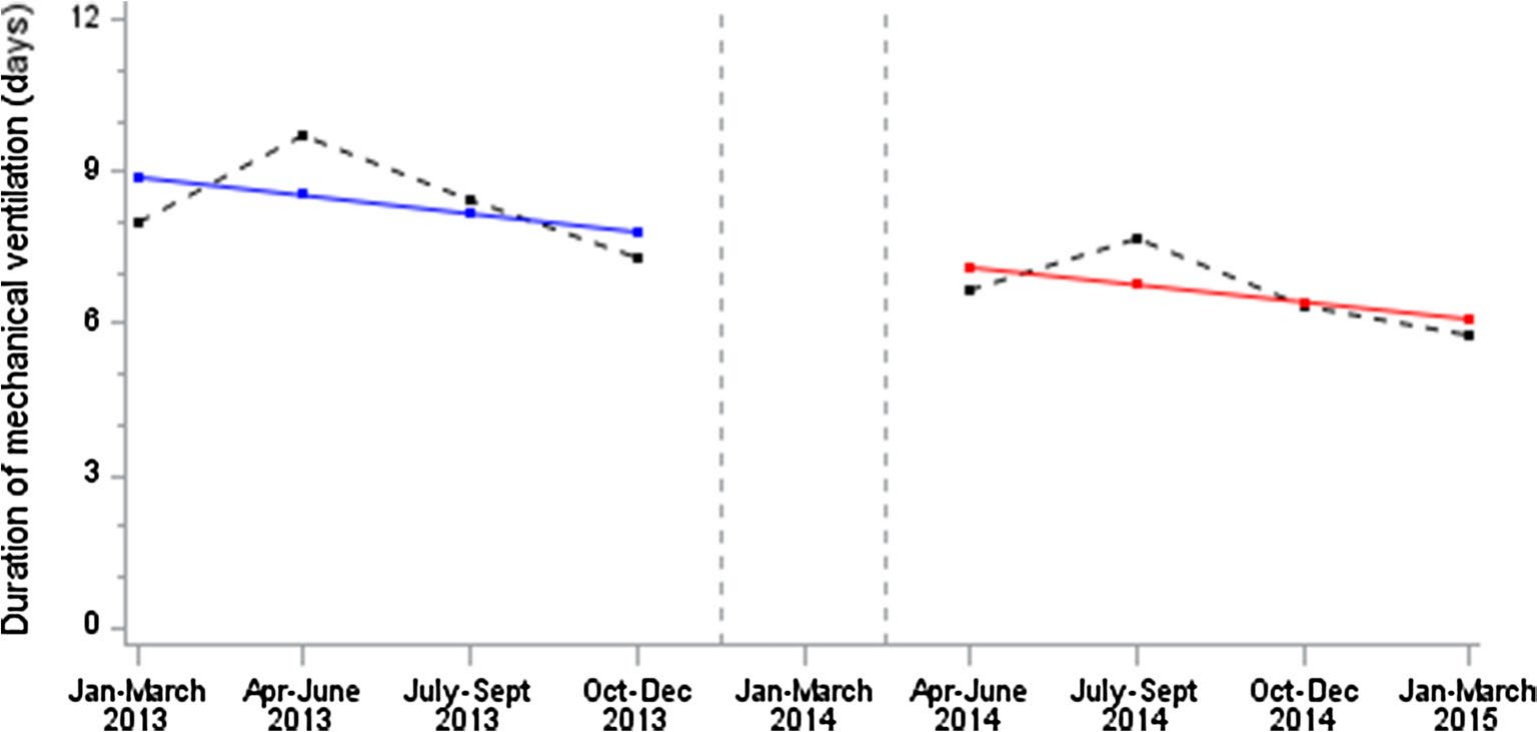
Duration of mechanical ventilation over time. Observed data are *black dots,* and predicted data with segmented regression analysis are *blue* for pre-implementation and *red* for post-implementation. *Vertical dashed lines* represent the 4-month implementation period

The results from segmented regression analysis are presented in Table 3 and Fig. 4. Before the implementation of the nurse-driven sedation protocol, the duration of the mechanical ventilation decreased each semester by 0.35 ± 0.42 days. The intervention had no significant immediate effect on the duration of the mechanical ventilation (*p* = 0.923 for immediate change after the implementation) and led to a non-significant change in trend over semesters following the implementation (*p* = 0.933 for change in trend after the implementation). Finally, the duration of the mechanical ventilation decreased significantly by 0.35 ± 0.10 days per semester over all the study period (*p* = 0.012, Fig. 4).

**Table 3:** Analysis of changes in the level and trend in outcomes after implementation of the nurse-driven sedation protocol

Outcomes	Pre-implementation period From 1 January 2013 to 31 December 2013 (*n* = 104 children)		Post-implementation period From 1 May 2014 to 31 March 2015 (*n* = 93 children)			
	Overall trend before the implementation of protocol^a^	*p* value	Immediate change after the implementation of protocol^b^	*p* value	Change in trend after the implementation of protocol^c^	*p* value
Duration of mechanical ventilation (days)	−0.35 (0.42)	0.450	0.17 (1.64)	0.923	−0.05 (0.60)	0.933
PICU length of stay (days)	−0.71 (0.90)	0.476	6.56 (3.49)	0.134	−1.07 (1.28)	0.450
Sufentanil						
Daily dose (μg/kg/day)	0.19 (0.31)	0.574	0.38 (1.22)	0.770	−0.21 (0.44)	0.668
Duration of administration (h)	−3.31 (4.79)	0.528	−1.42 (18.6)	0.943	−1.32 (6.78)	0.855
Midazolam						
Daily dose (mg/kg/day)	−0.12 (0.18)	0.541	0.04 (0.69)	0.955	0.02 (0.25)	0.944
Duration of administration (h)	0.27 (3.59)	0.944	−13.4 (13.9)	0.389	−3.39 (5.07)	0.541
Ketamine						
Daily dose (mg/kg/day)	1.11 (2.96)	0.727	1.97 (11.5)	0.872	−3.06 (4.19)	0.506
Duration of administration (h)	7.06 (21.2)	0.756	−4.82 (82.1)	0.956	−17.2 (30.0)	0.598

Negative numbers represent a decline, and inversely, positive numbers represent an increase in outcomes values

^a^Mean change (standard deviation in brackets) of each outcome per semester before the implementation of the protocol

^b^Expressed as a mean change (standard deviation in brackets) of each outcome immediately after implementation of the nurse-driven protocol compared with the pre-implementation period

^c^Mean change (standard deviation in brackets) of each outcome per semester after the implementation of the protocol compared with the pre-implementation period

Regarding secondary outcomes, we found no significant immediate level change or slope change after the implementation of the nurse-driven sedation protocol. Moreover, the PICU length of stay and the daily dose of sufentanil and ketamine were stable over time, whereas the daily dose of midazolam, and the duration of administration of sufentanil and midazolam decreased significantly over the study period (−0.10 ± 0.04 µg/kg/day per semester for the daily dose of midazolam, *p* = 0.051; 4.2 ± 1.1 h per semester for the duration of administration of sufentanil, *p* = 0.010; and −3.7 ± 1.0 h per semester for the duration of administration of midazolam, *p* = 0.010).

## Discussion

Guided sedation has been widely developed in recent years. However, to the best of our knowledge, there are no large-cohort studies describing a positive effect on the duration of mechanical ventilation in a paediatric population. Thus, we developed a sedation protocol and evaluated its influence on practices.

### Population

The median age of the population in each period was 2 and 3 years, respectively, which is similar to that of other studies [15, 18]. The mortality rate in our study population (17%) was higher than in our overall PICU population (5%). However, this higher mortality rate could be expected as ventilated patients are likely to be more severely ill, as illustrated by the high mean PELOD scores in both groups: 18.8 and 17.5.

### Population targeted by the protocol

We limited the nurses’ actions to the group “adequate sedation” with scores between 11 and 17. In case of deep sedation required for medical reasons, expected scores ranged from 7 to 11. In this setting, any modification of sedation or analgesia had to be validated by the physician. Recently, Gaillard-Le Roux et al. [30] showed in a paediatric study that a nurse-driven protocol was also applicable for deeply sedated patients, with lower target scores. Generalising the use of the protocol to all patient categories is certainly a good manner to enhance its acceptability and its efficacy.

### Impact on duration of mechanical ventilation

All recent large-cohort studies chose duration of mechanical ventilation as the primary outcome, but none were able to show a significant reduction in mechanical ventilation [15–18]. Except for the study by Curley et al., which was a randomised, controlled trial in children with ARDS, the other studies were designed as prospective or retrospective before and after studies.

Either with nurse-driven sedation protocol or with daily interrupted sedation, obtaining significant reduction in mechanical ventilation appeared to be difficult in PICU. This may be linked to various factors which may contribute to delay mechanical ventilation weaning, especially in younger children: difficulties to evaluate pain and comfort, fear of extubation failure, hemodynamic complications with sedation drugs, and occurrence of withdrawal symptoms. A wide weight range of children is admitted in PICUs (i.e. infants weighting less than 10 kg as well as children and teenagers weighing sometimes more than 80 kg), and there are probably important differences, especially pharmacologic ones, according to the age and to the reason for admission. A recent French paediatric study showed in a subgroup analysis a shorter length of mechanical ventilation among children older than 12 months after implementation of the protocol [30]. The only large RCT published on this subject [18] included patients with ARDS only, who present specific characteristics: long respiratory recovery, use of deep sedation and neuromuscular blockades; in consequence, their results could not be generalised to the overall PICU population. However, we did not performed subgroups analysis as the power of our study did not allow to. Future larger studies could bring interesting results on the use of guided sedation and analgesia in general population in PICUs and in specific groups of patients according to age or admission diagnosis.

### Impact on drug prescriptions

After implementation of a nurse-driven sedation protocol, Keogh et al. [17] showed a reduction in the duration of morphine administration of 19 h, and Neunhoeffer et al. [15] described a significant reduction in benzodiazepine cumulative doses after application of the protocol. Deeter et al. [16] compared patients treated under a nurse-driven protocol to a historic cohort and highlighted that the protocol enabled the duration of sedative drugs to be reduced from 7 to 5 days. For our part, we showed a downward trend in the duration of sufentanil administration and daily doses of ketamine in the post-implementation period, results not confirmed by segmented regression analysis.

### Impact on withdrawal symptoms

In children, withdrawal symptoms occurred frequently when the analgesics and sedatives are stopped. The incidence of withdrawal reported in the literature varies from 10% [31], 37% [4], and even 48% [32]. Withdrawal symptoms are linked to discontinuation of either opioids or benzodiazepines and result from central nervous system irritability, gastrointestinal dysfunction, and autonomic nervous system dysfunction. The WAT-1 score is now used routinely in our PICU to detect occurrences of withdrawal [25, 26]. In their before and after study design including 165 and 172 patients, respectively, Neunhoeffer et al. [15] showed that the use of a sedation protocol enabled significant reductions in the incidence of withdrawal symptoms from 23.6% before and 12.8% after implementing the protocol (*p* = 0.005). In our population, withdrawal symptoms tended to decrease without reaching significance.

### Impact on evaluation of sedation and analgesia

One of our main positive results was the improvement in sedation and analgesia evaluation. A fear could be that guided sedation led to insufficient sedation. In their randomised clinical trial, Curley et al. [18] described more reports of agitation and elevated pain scores in the group under guided sedation. In our experience, we recorded no increase in the number of COMFORT-B scores >17 or in unplanned endotracheal extubations.

### Limitations of the study

Our study has several limitations. First of all, it was not a randomised controlled trial but a “before and after” observational study performed within a single PICU, as majority of precedent studies [15, 17]; thus, results could possibly be influenced by temporal trend rather than to the protocol efficiency itself. This phenomenon was well described when interventions diffuse into widespread practice in an uncontrolled way while studies evaluating them are under way; for example, implementation of guidelines could have a positive impact before their official publication [33, 34]. Nevertheless, there was no change in general treatment or ventilator care strategies during the two study periods. Even so, we cannot exclude that implementation of the protocol could have made physicians and nurses more careful and aware of the importance of reducing unnecessary sedation and ventilation. However to limit this bias, we performed a segmented regression analysis.

Oversedation persisted in the post-implementation period, and this is probably one of the major limitations of our work. The proportion of scores <11 per patient found in our study is more important than reported in literature [5]. This result could be explained by the fact that any patient receiving more than 24 h of sedation was included in our study, even patients for whom the protocol was not applied, like patients under deep sedation for medical reasons and patients under neuromuscular blockade. Compared to other studies, patients under neuromuscular blockade were not excluded from our study; the use of this treatment was often limited in time, mainly in patients with respiratory disease (i.e. ARDS), and the protocol was successfully applied after the neuromuscular blockade discontinuation. Even if the proportion of patients receiving neuromuscular blockades is comparable between the two groups, the assessments under these medications may partly explain the high proportion of oversedation observations.

Another point to explain oversedation was probably the imperfect compliance to the protocol and the reluctance of nurses to decrease the drugs especially in a calm and comfortable patient. Thus, lack of compliance could have a major negative impact on our results. Unfortunately, in this study, the protocol compliance was not assessed; it should be the aim of a further study; for example, it could be interesting to analyse the discordances between the COMFORT-B scores and the nurses’ actions.

Implementing the protocol corresponded with major changes in practice and considerable modification of the medical staffs’ and nurses’ roles, giving more responsibility and independence to the PICU nurses. In previous studies, nurses seemed the best able to evaluate the patient’s sedation state and to adapt the therapeutics [17]. We certainly could have enhanced the quality of our work with realising a quality evaluation of our protocol. Keogh et al. [17] carried out a staff survey after their protocol implementation; they demonstrated that negative points were 1—difficult comprehension of the protocol, 2—need for full concentration with attention to details, 3—practice to become familiar with, and 4—lack of medical leadership. In our study, we should wonder if the 4-month period of adaptation was long enough, if all the nurses had received clear information and if sufficient continuous training was done after the implementation period.

Recently, Deeter’s team re-evaluated the effect of a nurse-driven protocol 5 years after its implementation [35]. Whereas in 2008, just after the protocol implementation, they showed a reduction in the median total sedation days from 7 to 5 days, in 2013, 5 years later, they obtained longer duration of total sedation days, mechanical ventilation, and PICU length of stay. The authors involved lack of routine feedback and of ongoing education programme, loss of interest, and redirection of priorities to other clinical concerns. With our guided sedation protocol, we replaced sedation and analgesia at the centre of our care; in the future, it will require good communication and in-service training in order to ensure long-term adherence to the protocol and to sustain efforts over time.

Lastly, we unfortunately did not assess the impact of our nurse-driven sedation protocol on outcomes such as delirium and post-traumatic stress disorder (PTSD) which are important issues for patients admitted to intensive care units. Indeed, the reported prevalence of paediatric delirium in PICU varies between 4 and 29% [36] and may be attenuated by better sedation and analgesia management. Regarding PTSD, a recent review [37] reported an incidence in adults of 17–34% one year after intensive care unit discharge. Risk factors seemed to be anterior psychological disorders, benzodiazepine administration, and delirious memories facilitated by pain or agitation. In children, Colville et al. [38] questioned 102 children (7–17 years of age) three months after their hospital discharge, according to the “Children’s Impact of Event Scale” and found an incidence of PTSD of 28. Administration of opioids or benzodiazepines for more than 48 h was significantly associated with the existence of delirious memories. The risk of occurrence of PTSD was 3 times greater when delirious memories were related. In the future, it could also be relevant to evaluate whether or not guided sedation protocols could influence delirium prevalence and long-term psychological complications in children.

## Conclusion

We conclude that implementation of a nurse-driven sedation protocol was feasible and was accepted as standard practice in a PICU. Evaluation of sedation and analgesia with the COMFORT-B score was improved by the protocol. The total number of scores and, above all, the number of optimal scores were significantly increased after the protocol implementation. The duration of mechanical ventilation, duration of sufentanil administration, and daily dose of ketamine tended to decrease thanks to the use of the protocol. Withdrawal symptoms seemed to be less frequent in the post-implementation period. Because of the design of the study, we cannot confirm that the results are linked to the protocol implementation and not to temporal trends in practice.

### Additional files

**Additional file 1.** Duration of mechanical ventilation and PICU lenght of stay in pre- and post- implementation period after adjustment on reason for admission.

**Additional file 2.** Pre-post analysis of additional drugs (for withdrawal symptoms).

## Appendix: Interrupted time series analysis

We estimated the model:

$$ Y_{t} = \beta_{0} + \beta_{1} *Time_{t} + \beta_{2} *Intervention + \beta_{3} *TimeAfterIntervention + e_{t} $$

where:

- Y_t_ indicates the mean outcome at trimester t.
- *β*_0_ estimates the baseline level of the outcome at the beginning of the study period (1st trimester of the overall study period).
- “Time” is an indicator of time and covers the 9 trimesters of study (variable coded 1–9). The coefficient *β*_1_ on this variable captures the overall secular trend in means outcomes over the study period.
- “Intervention” is a binary variable indicating the time periods in which the nurse-driven sedation protocol was in effect. Intervention is coded 0 for trimesters before the implementation (before January, 2014) and 1 for trimesters after the implementation of the nurse-driven sedation protocol (after January, 2014). The coefficient *β*_2_ on this variable is interpreted as the immediate change in the level of the outcomes.
- “TimeAfterIntervention” is coded 0 for trimesters before the implementation (before January, 2014) and sequentially numbers time periods after implementation (1–4). The coefficient *β*_3_ on this variable captures the continuing effect of the nurse-driven sedation protocol that is the slope of the change in successive time periods.

## Abbreviation

PICU: paediatric intensive care unit
COMFORT-B score: COMFORT behaviour score
ARDS: acute respiratory distress syndrome
PELOD: paediatric logistic organ dysfunction
WAT-1: withdrawal assessment tool 1
PTSD: post-traumatic stress disease
