# Supplementary material for: Implementation and evaluation of a paediatric nurse-driven sedation protocol in a paediatric intensive care unit
Source: Ann Intensive Care. 2017 Mar 24;7:36. doi: 10.1186/s13613-017-0256-7 (PMC5366991; doi:10.1186/s13613-017-0256-7)
Supplement: Supplementary file 1 — Additional file 1. Duration of mechanical ventilation and PICU lenght of stay in pre- and post- implementation period after adjustment on reason for admission. [file 13613_2017_256_MOESM1_ESM.doc]

|  | **Pre-implementation**  **n=104** | **Post-implementation**  **n=93** | **p-value*** |
| --- | --- | --- | --- |
| **Duration of mechanical ventilation (days)**  Surgical cause  Mean (SD)  Median [Q1-Q3]  Medical cause  Mean (SD)  Median [Q1-Q3] | N=25  7.1 (6.2)  4.8 [2.8-9.5]  N=79  8.7 (7.6)  6.2 [4.0-10.8] | N=35  4.9 (4.4)  4.1 [2.0-5.7]  N=58  7.6 (6.0)  5.6 [3.3-9.3] | p=0.133 |
|  |  |  |  |
| **PICU length of stay (days)**  Surgical cause  Mean (SD)  Median [Q1-Q3]  Medical cause  Mean (SD)  Median [Q1-Q3] | N=25  11.1 (10.7)  6.8 [4.3-12.7]  N=79  12.9 (11.4)  9.3 [5.8-16.1] | N=35  13.0 (11.7)  9.3 [4.6-13.3]  N=58  11.5 (7.4)  9.9 [4.9-16.0] | p=0.784 |
