# Supplementary material for: Implementation and evaluation of a paediatric nurse-driven sedation protocol in a paediatric intensive care unit
Source: Ann Intensive Care. 2017 Mar 24;7:36. doi: 10.1186/s13613-017-0256-7 (PMC5366991; doi:10.1186/s13613-017-0256-7)
Supplement: Supplementary file 2 — Additional file 2. Pre-post analysis of additional drugs (for withdrawal symptoms). [file 13613_2017_256_MOESM2_ESM.doc]

|  | **Pre-implementation**  **n=104** | **Post-implementation**  **n=93** | **p-value** |
| --- | --- | --- | --- |
| **Total dose of clonidine, µg**  Mean (SD)  Median [range]  Q1-Q3 | n=10  92.0 ± 151.8  33.3 [2.4-504.9]  8.3-96.5 | n=6  25.6± 25.4  13.5 [2.5-59.3]  8.4-56.5 | 0.416 |
| **Total dose of hydroxyzine, mg**  Mean (SD)  Median [range]  Q1-Q3 | n=42  6.0± 15.5  1.3 [0.37-88.9]  0.87-2.60 | n=51  4.9 ± 6.8  2.5 [0.3-35.9]  0.7-6.5 | 0.244 |
| **Total dose of levomepromazine, mg**  Mean (SD)  Median [range]  Q1-Q3 | n=30  9.0 ± 13.3  4.5 [0.2– 56.6]  2.1– 9.5 | n=35  5.7 ± 5.9  3.7 [0.3-23.3]  0.9-9.9 | 0.389 |
| **Total dose of methadone, mg**  Mean (SD)  Median [range]  Q1-Q3 | n=27  1.7 ± 1.2  1.4 [0.1-4.4]  0.7-2.6 | n=16  1.3 ± 1.1  1.0 [0.2-4.0]  0.5-1.7 | 0.253 |
| **Total dose of pentobarbital, mg**  Mean (SD)  Median [range]  Q1-Q3 | n=21  49.1 ± 74.1  18.5 [3.6-317.5]  9.4-54.0 | n=18  40.8 ± 45.2  23.4 [2.1-161.5]  8.8-63.2 | 0.955 |
| **Total dose of clorazepate, mg**  Mean (SD)  Median [range]  Q1-Q3 | n=44  4.4 ± 4.3  2.9 [0.2-16.0  0.8-6.1 | n=40  4.7± 6.6  2.1 [0.2-35.3]  0.7-4.9 | 0.576 |

Table: Pre-post analysis of secondary drugs
